# The New Spine of Access to the Brain’s Secrets: Extracellular Vesicles from Cerebrospinal Fluid Liquid Biopsies in CNS Diseases and Blood–Brain Barrier Research

**DOI:** 10.1007/s12035-026-06074-6

**Published:** 2026-07-23

**Authors:** Isabeau Vermeulen, Yannick Parmentier, Jill Barber, Arnaud François, Loubna Chadli, Nicolas Provost, Emre M. Isin, Kayode Ogungbenro, Aleksandra Galetin, Amin Rostami-Hodjegan, Zubida M. Al-Majdoub

**Affiliations:** 1 Centre for Applied Pharmacokinetic Research, University of Manchester, Manchester, UK; 2 Institut de R&D Servier, Paris-Saclay Gif-Sur-Yvette, France; 3 Certara, Predictive Technologies, Sheffield, UK

**Keywords:** Liquid biopsy, CSF, Extracellular vesicles, CNS diseases, Blood–brain barrier

## Abstract

Liquid biopsy is emerging as a powerful approach for less invasive biomarker discovery, with extracellular vesicles (EVs) in cerebrospinal fluid (CSF) showing promise for the assessment of central nervous system (CNS) disorders without actual tissue biopsy and as a complement to imaging techniques. EVs carry molecular cargo such as proteins, nucleic acids, and lipids that mirror those at the tissue of origin, offering unique opportunities to quantify disease-related changes in biomarkers. Compared with plasma-derived EVs, those from CSF provide more direct insights into the CNS because of direct shedding of brain EVs to CSF and bypass of confounding factors involving entry to systemic circulation. Despite this potential, translation into clinical practice is limited by challenges such as low yields, purity concerns, and lack of standardized isolation protocols. Addressing these difficulties, alongside integrating multiomics approaches, will advance our understanding of EV molecular cargo and their functional roles in CNS diseases. Over time, CSF-derived EVs could become the new driver of precision medicine in neurology, offering biologic insight for both diagnostic and therapeutic applications. This perspective provides a critical evaluation of the current status of EV-based liquid biopsy in CSF and offers recommendations for future research and clinical translation of data from CSF-derived EVs, highlighting their potential to inform physiologically based pharmacokinetic (PBPK) models. This state-of-the-art article evaluates existing evidence and highlights key knowledge gaps.

## Introduction

Liquid biopsies have emerged as a powerful approach for biomarker discovery and diagnostics, providing a relatively less invasive alternative to conventional tissue biopsies. By analyzing biofluids, such as urine, plasma, and cerebrospinal fluid (CSF), they enable repeated sampling and real-time monitoring of disease progression. Importantly, liquid biopsies facilitate the early detection of brain tumors and central nervous system (CNS) diseases, often before clinical symptoms arise, which is critical for improving outcomes [1–3]. This minimally invasive approach is particularly valuable for CNS diseases, where traditional diagnostic methods such as brain tissue biopsy are highly invasive and practically unfeasible [4, 5]. In this perspective, we provide a critical overview of current knowledge on CSF-derived EVs in CNS diseases and BBB research, rather than a systematic review following a predefined protocol. Our goal is to integrate recent findings across oncology, neurodegeneration, and BBB biology, identify methodological bottlenecks in CSF-EV isolation and validation, and highlight areas where targeted experimental and clinical work is needed.

Much of the focus with liquid biopsy has come from neuro-oncology. Tumor-derived components, such as circulating tumor cells (CTCs), cell-free DNA (cfDNA), and circulating tumor DNA (ctDNA), can be detected in plasma, urine, and CSF and have been used to monitor cancer progression and therapeutic response [6, 7]. Elevated cfDNA levels in glioblastoma and correlations between ctDNA and tumor glioma volume or grade further support their diagnostic value [8–11]. Integrating molecular features, including DNA methylation and proteomic profiles, into CSF analysis can also help distinguish gliomas from other brain tumors [12]. These findings demonstrate that liquid biopsy can support glioma diagnosis, track progression, and guide treatment in a less invasive manner. Numerous studies using plasma and CSF reinforce its value not only for biomarker discovery but also for improving our understanding of tumor biology [13–17]. However, liquid biopsies can be used beyond cancer diagnosis to detect biomarkers for CNS diseases, including neurodegenerative conditions [18]. A well-established protein marker is neurofilament light chain (NfL), a sensitive indicator of axonal injury. NfL levels rise in several conditions, such as amyotrophic lateral sclerosis (ALS), multiple sclerosis (MS), and dementia including Alzheimer’s disease (AD) [19, 20]. Another frequently studied marker is glial fibrillary acidic protein (GFAP) which reflects reactive astrogliosis and shows diagnostic value in disorders including MS, AD, and multiple system atrophy (MSA) [21, 22]. Additional biomarkers include α-synuclein in Parkinson’s disease and amyloid-beta/tau in AD [23–25]. More recent work has expanded the biomarkers spectrum to proteins linked to metabolic pathways, inflammation, and synaptic function [26]. Together, these examples highlight the capacity of liquid biopsies to reveal protein changes that reflect core pathological processes across neurodegenerative conditions.

Beyond proteins, other biomolecules such as nucleic acids, lipids, and metabolites also serve as biomarkers for the CNS [27, 28]. These markers not only inform on disease mechanisms and progression but also provide insight into blood–brain barrier (BBB) integrity. Changes in these molecules may indicate BBB dysfunction or altered permeability, which is crucial in various CNS disorders and their drug treatment. The primary focus of this perspective is on CSF-derived EVs, with plasma EVs discussed only as a comparative reference.

## Extracellular Vesicles in CSF: Biology and Relevance

**Fig. 1:**
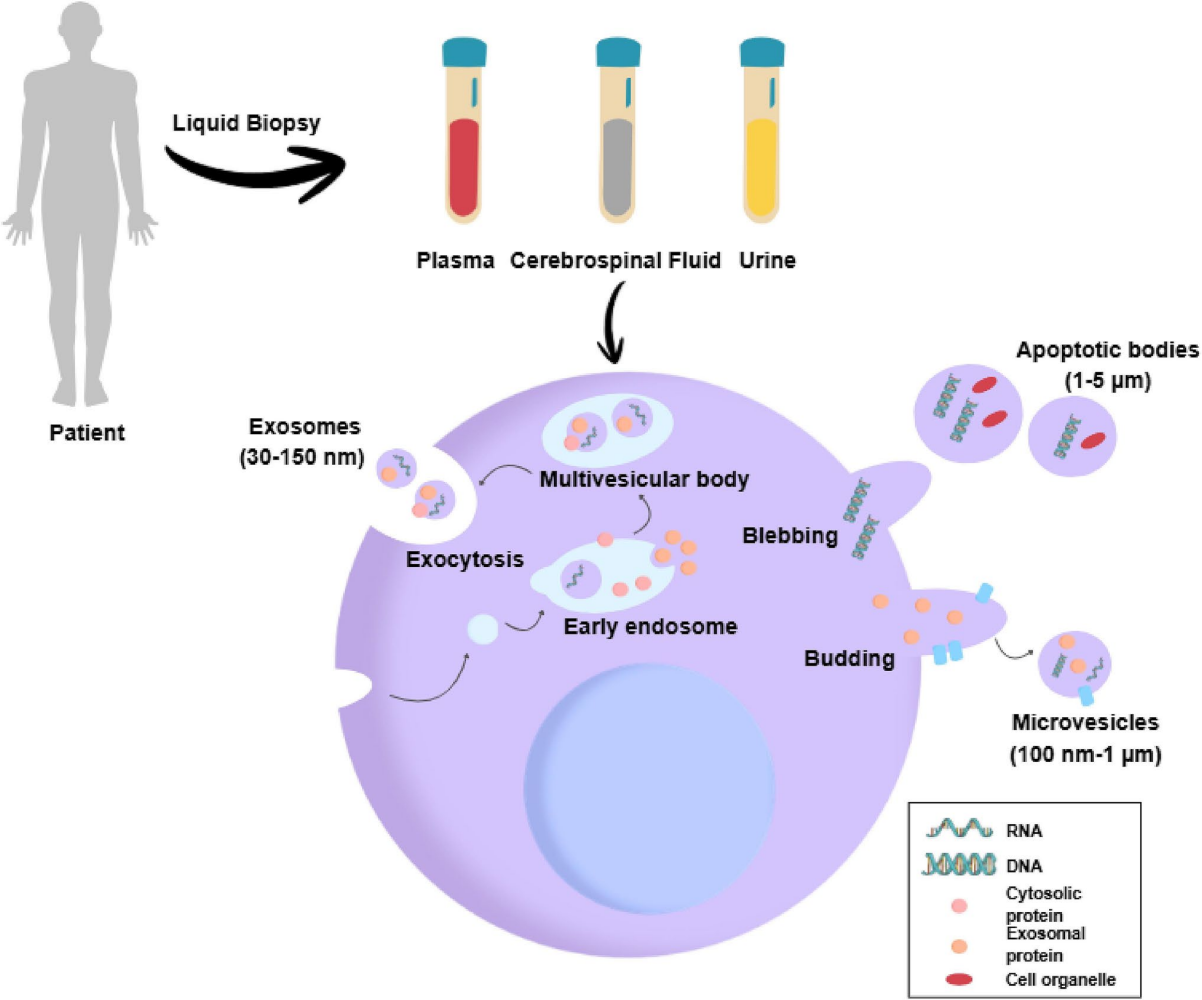
Overview of liquid biopsy sources (plasma, cerebrospinal fluid, and urine) and extracellular vesicle biogenesis

EVs present in CSF are membrane-bound particles released by cells within the central nervous system (CNS), reflecting ongoing molecular and cellular processes in the brain. They carry proteins, nucleic acids, and metabolites, acting as mediators of intercellular communication and offer significant potential as biomarkers in liquid biopsies [29]. Compared with other biofluids, CSF contains a relatively low abundance of EVs, with concentrations reported to be several orders of magnitude lower than those found in plasma. Studies have shown that CSF contains approximately 500–1000-fold fewer total nanoparticles and up to 10,000-fold fewer tetraspanin-positive EVs than plasma or serum [30, 31]. In addition, CSF contains substantially lower concentrations of soluble proteins, with total protein levels reported to be approximately 200–400-fold lower than those in plasma [32]. Although EVs from other biofluids such as urine have also shown diagnostic potential, this review focuses on EVs from CSF and compare these with plasma EVs. Based on their biogenesis and size, EVs identified in CSF can be categorized into (i) microvesicles (MVs), (ii) apoptotic bodies, and (iii) exosomes (ISEV 2023 guidelines (MISEV2023)) (Fig. 1). Apoptotic bodies, resulting from cell death, reflect cellular turnover, whereas MVs bud directly from the plasma membrane. Exosomes (30–150 nm diameter) originate from the endosomal system [33–36]. Within the CNS, astrocytes, oligodendrocytes, neurons, and microglia all release EVs that regulate diverse physiological processes, highlighting the complexity of EV biogenesis and their potential roles in intercellular communication [37].

CSF EVs carry diverse cargo and contribute to tissue homeostasis, influencing cell proliferation, immune response, neuron protection, and overall neuronal function [38]. CSF-derived EVs transport proteins and nucleic acids [39, 40], making them promising biomarkers for CNS diseases. For instance, oligodendrocyte-derived EVs are rich in myelin proteins and lipids and they carry molecules that regulate myelin production and support neuronal health [40]. CSF-derived EVs can also boost neuronal resistance to oxidative stress by delivering protective proteins, while EVs released by cortical neurons participate in synaptic plasticity. EVs secreted by microglia have been linked to energy metabolism pathways [41], whereas neuron-derived EVs are involved in a broad range of biological functions, including synaptic function [41, 42].

In the peripheral nervous system, Schwann cell EVs support axons and facilitate regeneration after nerve injury [43], and although they are not directly sampled in CSF, they illustrate the broader relevance of EV-mediated communication in the nervous system. Within the CNS, EV secretion is tightly regulated by neurotransmitters, so secretion patterns can mirror the pathogenic processes originating in brain cells [40, 44]. Understanding how CSF-derived EVs mediate these functions and how they change in disease will clarify key aspects of CNS biology. EVs as biomarkers are increasingly studied in AD [45–47], Parkinson’s disease [44, 48, 49], Lewy body dementia [50], amyotrophic lateral sclerosis (ALS) [51], traumatic brain injury [37], and viral-mediated neurological diseases [52]. Among these, CSF-derived EVs are of particular interest due to their close association with CNS pathology. Beyond biomarker potential, EVs are now considered to actively contribute to disease progression: In AD, neuron-derived EVs carry β-amyloid and phosphorylated tau, facilitating aggregate propagation and neuronal dysfunction, while in Parkinson’s disease, EVs transport aggregated α-synuclein and may promote disease spread [53]. This disease propagation may reflect EVs’ intrinsic role in intercellular communication and their unique capacity to cross the BBB [52]. These insights point to EV-mediated pathological cargo transfer as a potential therapeutic target in neurodegenerative disease. Finally, although many studies focus on exosomes, current isolation and validation protocols often cannot distinguish exosomes from other EVs subtypes and therefore use the broader term “EVs,” in line with recommendations from the Minimal Information for Studies of Extracellular Vesicles [54]. This perspective will provide an overview of liquid biopsy–derived EVs in biomarker discovery, with a focus on CSF-derived EVs and their connection to the BBB and will discuss current challenges regarding the isolation and validation of EVs as well as future diagnostic, prognostic and mechanistic contexts.

## From Systemic Baseline to CNS Specificity: Comparative Protein and RNA Cargo of Plasma and CSF-EVs

Because EVs mirror the molecular state of their parent cells, EV-associated cargo can provide real-time insights into cellular processes, including those within the CNS. Liquid biopsy approaches leveraging EV-associated proteins and nucleic acids have been increasingly explored. Among these, plasma-derived EVs are the most extensively studied due to their accessibility and minimally invasive collection [55–57]. The convenience of plasma sampling is a major advantage, but the real value lies in the measurable levels of proteins, nucleic acids, and other biomarkers specifically encapsulated within plasma-derived EVs, which may differ from soluble, free-circulating biomarkers in the same fluid. This raises two critical questions for translation: (I) How specific are these EV-associated signals: are they sufficiently unique to serve as reliable indicators of disease, or might they be influenced by systemic factors such as lifestyle or medication? (II) How do EV-derived biomarker concentrations reflect the originating tissue? Plasma EV liquid biopsy has broad sensitivity, particularly for detecting systemic involvement in CNS diseases and adding diagnostic signal beyond the CNS. However, one must consider whether this broader sensitivity could lead to overdiagnosis or detection of clinically insignificant EV-associated biomarkers, especially in diseases with overlapping symptomatology. Thus, emphasis should be placed on clinically relevant EV-associated readouts, rather than their presence in plasma-derived EVs. For example, Dutta et al. (2021) investigated immunoprecipitated EVs from plasma using neuronal (L1CAM) and oligodendroglial (MOG) markers and showed that EV-associated α-synuclein levels in these distinct EV populations could distinguish multiple system atrophy (MSA) from Parkinson’s disease. This distinction is consistent with the underlying pathology, where α-synuclein aggregates primarily accumulate in neurons in Parkinson’s disease and in oligodendrocytes in MSA. Without such enrichment strategies, plasma-derived EV analyses risk conflating signals from multiple cell types, potentially obscuring disease-specific signatures. Recent studies have highlighted the potential of both plasma-derived EVs and CSF-derived EVs as sources of protein and RNA biomarkers across different CNS diseases, as illustrated in Table 1. Where indicated, Table 1 includes soluble biomarkers measured in CSF (i.e., not EV-associated) for comparison alongside EV-based measurements. A key advantage of plasma EV-associated biomarkers is their accessibility compared to CSF, making them attractive candidates for less invasive diagnostics. However, while initial findings suggest diagnostic relevance, most studies of EV-associated biomarkers remain at the exploratory stage.

**Table 1:** Plasma-derived and CSF-derived extracellular vesicle–associated and soluble biomarkers in CNS diseases: current evidence

Disease	EV biomarker	Biofluid source	EV-associated	Diagnostic/prognostic role	Evidence in CSF-derived EVs	References
EV-associated biomarkers						
Glioblastoma	Elevated EV concentration	Plasma	Yes	Potential diagnostic marker	Not tested	[4, 62, 63]
Medulloblastoma (metastatic)	Matrix metalloproteinase-2 (MMP-2) enriched exosomes	Plasma	Yes	Promotes tumor cell invasiveness	Not tested	[58]
Multiple sclerosis	50 + proteins linked to complement/coagulation/wound healing	CSF	Yes	Potential diagnostic marker	Exploratory	[5]
Parkinson’s disease/multiple system atrophy	α-Synuclein	Plasma	Yes	Potential differential diagnostic marker for Parkinson’s disease vs MSA	Exploratory	[64, 65]
Amyotrophic lateral sclerosis/frontotemporal dementia	TAR DNA-binding protein 43 (TDP-43), three-repeat/four-repeat (3R/4R) tau isoforms	Plasma	Yes	Potential diagnostic marker	Exploratory	[60]
Alzheimer’s disease	Differential phosphorylated tau content	CSF	Yes	Potential diagnostic marker	Exploratory	[66]
Alzheimer’s disease	S100 calcium-binding protein A8 (S100A8)	Plasma	Yes	Proposed diagnostic marker	Not tested	[59]
Alzheimer’s disease	Complement component 1q (C1q) and differentially expressed proteins	CSF	Yes	Implicated in immune response	Exploratory	[67]
Alzheimer’s disease	Altered cathepsin B levels	CSF	Yes	Potential diagnostic marker	Exploratory	[68]
Traumatic brain injury	Elevated olfactory receptors	CSF	Yes	Potential biomarker	Exploratory	[69]
Soluble biomarkers (non-EV)						
Multiple CNS disorders (multiple system atrophy, Alzheimer’s disease, amyotrophic lateral sclerosis)	Glial fibrillary acidic protein (GFAP)	Plasma/CSF	No	Diagnostic marker of reactive astrogliosis	Soluble (non-EV) **MS:** AUC 0.74–0.82, Spec up to 100% in active phases [70–72] **AD:** AUC 0.72–0.77, Sens 70–80%, Spec 75–88% [73–75] often outperformed by plasma GFAP [76, 77] **FTD:** AUC ~ 0.80 [76]	[22, 78, 79]
Multiple CNS disorders (Alzheimer’s disease, Parkinson’s disease, amyotrophic lateral sclerosis, multiple system atrophy)	Neurofilament light chain (NfL)	Plasma/CSF	No	Diagnostic marker for axonal damage and neurodegeneration	Soluble (non-EV) **MS:** AUC 0.85–0.93 [70] **AD:** AUC 0.84–0.87, Sens 78–85%, Spec 75–82% [73] **FTD:** AUC 0.86–0.93 for [80] **ALS:** AUC 0.95–0.99, Sens 91–97%, Spec 91–96% [81]	[82–85]
Alzheimer’s disease	Amyloid-beta 42/40 ratio (Aβ42/40)	Plasma/CSF	No	Established diagnostic marker	Soluble (non-EV) **AD:** AUC 0.90–0.97, Sens 85–94%, Spec 88–96% [73] **MCI:** AUC 0.85–0.92 for 3–5 year conversion to AD [86]	[83, 86–90]

EV;extracellular vesicle, CSF; cerebrospinal fluid, MCI; mild cognitive impairment, AD; alzheimer's disease; MS; multiple sclerosis, FTD; frontotemporal dementia, ALS; amyotrophic lateral sclerosis, AUC; area under the curve, sens; Sensitivity, spec; specificity

Notably, in oncology, plasma EVs not only reflect tumor burden but may also actively contribute to disease progression, as seen in metastatic medulloblastoma [58]. This dual role raises both opportunities (biomarkers) and considerations (functional effects). In neurodegeneration, EVs carrying S100A8, TDP-43, and tau isoforms offer promising molecular signatures, but further validation is needed to establish specificity and sensitivity of these EV-associated markers across different patient populations [59, 60]. Ultimately, the exploratory nature of these peripheral markers also underscores the necessity of CSF-focused EV research, as CSF-derived EVs provide a more direct and concentrated reflection of the neural microenvironment. Unlike blood-based assays, CSF provides a more direct insight into CNS pathology and CSF-derived EVs capture a proteomic signature that more accurately reflects central pathology without the confounding influence of the systemic circulation [61].

Although plasma-derived EVs have promising potential, CSF provides more precise and sensitive CNS-derived signals, making it a superior fluid for detecting neurological biomarkers in most cases [91–93]. As detailed in Table 1, CSF GFAP demonstrates variable diagnostic accuracy across the neurodegenerative and neuroinflammatory spectrum. Peak specificity is observed during active phases of MS (Spec up to 100%) [70], while its utility in AD remains moderate (AUC 0.72–0.77) and it is notably outperformed by plasma-based measurements [76, 83]. Furthermore, its diagnostic value in FTD appears largely restricted to specific genetic cohorts, particularly progranulin mutation carriers (AUC ~ 0.80) [76]. Importantly, in all of these contexts, GFAP refers to its soluble CSF form rather than an EV-associated protein, as CSF GFAP is primarily a marker of astrocytic injury and not a validated extracellular vesicle cargo. Similarly, NfL, a well-established marker of neuronal health and axonal damage, consistently shows higher concentrations in CSF compared to plasma, illustrating how peripheral measurements can be diluted by the systemic circulation. As with GFAP, CSF NfL is also predominantly measured as a soluble protein rather than an EV-associated biomarker, reflecting axonal injury rather than vesicle-encapsulated signaling. As detailed in Table 1, this robust CSF signal translates to high diagnostic accuracy in ALS [81] and a high diagnostic yield in distinguishing FTD from primary psychiatric mimics [80]. In MS and AD, these metrics primarily reflect the velocity of neuroaxonal collapse and long-term disability progression rather than acute diagnostic status [70, 73]. The final validated biomarker is the Aβ42/40 ratio, the gold standard for identifying Alzheimer’s disease. By correcting for total amyloid production, it achieves superior accuracy over Aβ42 alone (Table 1). This marker is also measured as a soluble peptide fraction in CSF. By extension, CSF-derived EVs may offer greater sensitivity for brain-specific molecular changes than plasma-derived EVs. Numerous studies have explored further the potential of CSF-derived EVs (i.e., vesicle-encapsulated proteins and RNAs, distinct from soluble CSF biomarkers such as GFAP, NfL, and Aβ peptides) as novel biomarkers across CNS diseases (Table 1). While some biomarkers, like the previously described GFAP, NfL, and Aβ42/40 ratio, are already established in their soluble form, others remain at an exploratory stage in the context of CSF-derived EVs. The identification of immune-related proteins and olfactory receptors in CSF-derived EVs further highlights their potential to uncover distinct pathophysiological mechanisms in a range of CNS disorders. Despite encouraging findings, clinical translation remains challenging. A major limitation is the lack of standardized protocols for isolating EVs, which leads to variability and limits reproducibility across studies. This challenge is particularly important for clinical implementation, where consistency and robustness are essential. To address these issues, ongoing efforts such as the International Society for Extracellular Vesicles (ISEV) task forces are working to develop and harmonize standardized protocols from CSF, urine, and plasma [54, 94–96]. In addition, the relatively rapid CSF turnover of 4–5 h complicates interpretation of EV-derived biomarkers, as their concentrations may be influenced by both secretion and clearance dynamics [97]. Ultimately, the clinical utility of these biomarkers depends on whether they reflect true brain pathology or systemic noise. This distinction applies differently to soluble CSF biomarkers versus EV-associated biomarkers: Soluble markers (e.g., GFAP, NfL, Aβ42/40) reflect passive release from injured or degenerating CNS tissue, whereas EV-associated biomarkers represent actively secreted vesicular cargo that retains cell type–specific molecular information. This distinction is entirely governed by the transport dynamics and integrity of the blood–brain barrier (BBB).

## CSF-Derived EVs and Their Connection to the BBB

This section focuses specifically on how CSF-derived EVs interact with and report on BBB and blood–CSF barrier dysfunction and how these relationships influence biomarker interpretation. Beyond serving as static biomarkers, these CSF-derived EVs act as dynamic indicators of the BBB status, reflecting the physiological and pathological state of the neurovascular unit. The BBB forms a selective interface that preserves CNS integrity by regulating the transfer of molecules from the circulation into the brain. The BBB is primarily formed by tightly connected brain endothelial cells, supported by astrocyte end-feet and pericytes, which together maintain barrier integrity and homeostasis [98]. In parallel, the blood–CSF barrier at the choroid plexus consists of fenestrated capillaries and a monolayer of epithelial cells joined by tight junctions, which actively secrete CSF into the ventricular system and regulate the exchange of solutes between blood and CSF [99]. The relationship between CSF-derived EVs and the BBB is defined by a bidirectional exchange: These vesicles function as diagnostic indicators of endothelial status or as active mediators of pathophysiological modulation [100–102]. Mechanistically, as CNS-derived vesicles undergo transcellular flux across the neurovascular unit, they incorporate membrane-associated components, providing a molecular snapshot of the cell of origin protein composition. This offers a distinct advantage over soluble biomarkers; while free proteins are subject to rapid proteolytic clearance, the EV lipid bilayer sequesters its cargo, preserving the concentration and stoichiometry of signaling molecules [100, 102, 103]. In various contexts, EV cargo can modulate endothelial function, promote inflammation, degrade tight junctions, or otherwise increase permeability thereby contributing to BBB disruption [46, 55, 63, 104–106]. CSF-derived EV composition is further shaped by CSF secretion and turnover (with a turnover time on the order of several hours) and by clearance pathways such as arachnoid granulations and paravascular/glymphatic flow, which together determine EV residence time within the CSF compartment [107]. Furthermore, compared to plasma-derived EVs, which are dominated by systemic signals from peripheral tissues, CSF EVs offer a localized readout of the CNS microenvironment. As a result, CSF EVs integrate signals from both the BBB and the B-CSF barrier, filtered through CSF flow dynamics, whereas plasma EVs primarily reflect systemic influences and BBB-mediated transport [61, 108]. This proximity allows for the detection of subtle changes in barrier-specific markers, such as claudin-5 or PECAM1, before they are diluted into the systemic circulation [109, 110]. This dual role as a biomarker of BBB dysfunction and a therapeutic vehicle highlights the growing significance of EVs. Table 2 summarizes specific EV-associated signatures, including membrane-bound proteins, encapsulated cargo, and vesicular metrics, that reflect changes in BBB integrity. Unlike independent soluble proteins in the CSF, these markers are characterized by their physical association with the vesicle, which enables the targeted mechanistic effects on barrier permeability described in the table.

**Table 2:** Extracellular vesicles and their role in blood–brain barrier disruption

Disease	EV-derived readouts	Type	Effect on BBB	Reference
Glioblastoma	Semaphorin3A (Sema3A)	EV surface-bound protein	Disrupts endothelial barrier	[105]
Glioblastoma	VEGFA	E-encapsulated/membrane-bound	Increases BBB permeability by downregulating claudin-5 and occludin	[106]
Glioblastoma	Total EV concentration	Vesicular biogenesis metric	Indicates BBB leakage	[63]
Alzheimer’s disease	Exosomal morphological profile	Intravesicular cargo	Reduces vascular endothelial cadherin expression, weakening BBB integrity	[46]
Multiple sclerosis	Endothelial-derived EV subsets	Membrane-defined subpopulations	Small EVs (sEVs) are protective; large EVs (lEVs) disrupt BBB integrity and enhance T cell migration	[55]
Multiple sclerosis	CNS-specific shedding rate	Origin-specific flux (quantified from EV markers in CSF)	Correlates with BBB permeability changes (MRI^a^-confirmed)	[55]

^a^MRI: magnetic resonance imaging

In addition to carrying cargo from CNS parenchymal cells, EVs that interact with or traverse the BBB and B-CSF barrier are likely to incorporate endothelial and barrier-associated components [109]. Consequently, EV populations detected in CSF and plasma may include vesicles derived from brain microvascular endothelial cells and choroid plexus epithelium, and not solely from neurons or glia [111]. Barrier-related markers such as claudin-5 (CLDN5) or PECAM1 on EVs could therefore provide informative readouts of barrier status and endothelial activation, but they also complicate the interpretation of “brain-derived” EV signatures by introducing a barrier component into the molecular profile [112, 113]. Distinguishing these specific signatures from the complex CSF matrix requires highly rigorous isolation and characterization protocols to ensure that the primary brain-specific cargo is not masked by systemic or endothelial noise.

## Challenges in Isolation and Characterization of EVs from CSF

CSF poses specific constraints for EV isolation that differ from those encountered with plasma. In contrast to plasma, CSF offers much smaller sample volumes and requires invasive collection, so EV isolation protocols must be optimized specifically for CSF to maximize recovery without compromising purity [114]. Although plasma-derived EVs are more suitable for routine clinical use, they lack sufficient sensitivity for CNS-specific marker quantification. CSF, by directly sampling the CNS compartment, provides more precise CNS-derived signals, but the combination of low volume, low EV abundance, and invasive sampling emphasizes the need for isolation workflows that are specifically optimized for low-volume CSF while preserving vesicle integrity and biomarker content.

## Overview of CSF-Derived EV Isolation Techniques

**Fig. 2:**
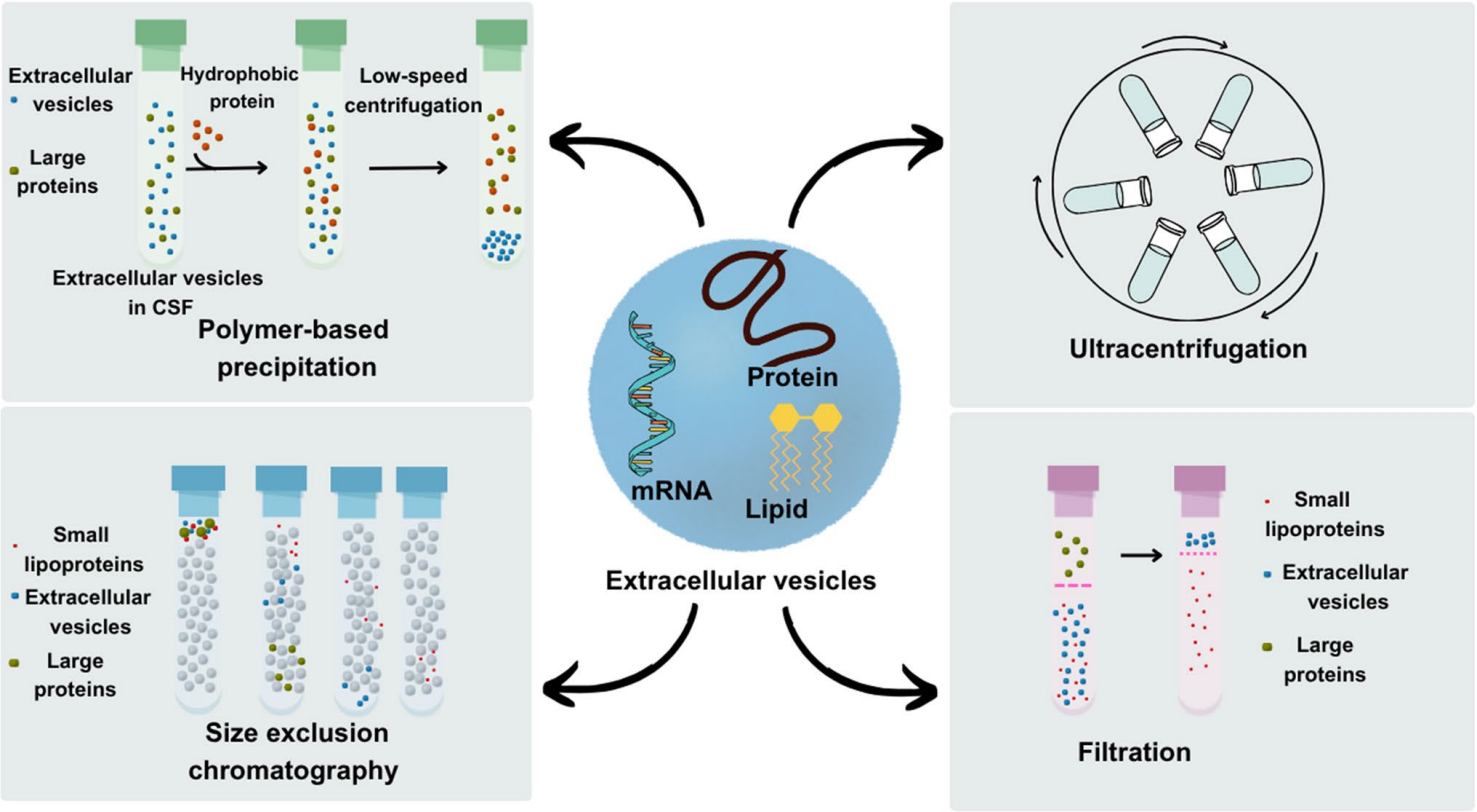
Overview of common methods for isolating extracellular vesicles from cerebrospinal fluid (CSF)

A variety of isolation methods have been applied to CSF-derived EVs, including ultracentrifugation (UC), polymer-based precipitation, size-exclusion chromatography (SEC), and ultrafiltration (UF), each with distinct advantages and limitations (Fig. 2, Table 3). UC is widely used approach and can provide relatively pure vesicle preparations, but it is time-consuming, requires specialized equipment, and may be less efficient when starting from limited CSF volumes. Polymer-based precipitation remains a popular and effective choice, with Invitrogen’s Total Exosome Isolation Reagent and Qiagen’s miRCURY Exosome kits frequently used for CSF in the literature [115, 116]. Both kits have shown comparable results, though one study favors miRCURY for CSF [117]. ExoQuick has also been reported as effective in several CSF studies, although co-isolation of non-EV proteins remains a limitation. SEC, another effective technique, has been implemented using Exo-Spin or Izon columns, yet no direct comparisons between these column types have been reported. Studies suggest that smaller pore sizes (e.g., 30–35 nm) yield better results by enhancing both purity and recovery by more effectively separating vesicles from abundant soluble proteins [118–120].

**Table 3:** Overview of EV isolation techniques from cerebrospinal fluid (CSF)

Technique	Characteristics	Advantages	Limitations	References
Ultracentrifugation (UC)^a^	High-speed centrifugation (≥ 100,000 × *g*)	Widely used; fast; no chemical reagents; relatively low cost	Low yield; risk of EV disruption; co-isolation of contaminants, large sample volumes	[111, 125, 126]
Polymer-based precipitation	PEG-based reagents precipitate EVs (e.g., ExoQuick, ULTRA)	High recovery; easy and fast; compatible with small volumes	Low purity; co-isolation of proteins and non-EV particles	[5]
Size-exclusion chromatography (SEC)	Separation based on size via porous resin columns	Gentle on EVs; improved purity (especially with 30-nm columns)	Lower yield; column performance depends on pore size and sample volume	[115, 119, 127]
Ultrafiltration (UF)	Membrane filtration based on molecular weight cutoff	Fast; no reagents required	Potential EV deformation; membrane clogging; variable recovery, lower yield	[128, 129]
Affinity capture/immuno-isolation	Use of antibodies or ligands targeting specific EV surface markers for selective capture	High specificity; allows enrichment of EVs from particular CNS cell types; compatible with downstream proteomics	Marker-dependent (requires known surface proteins); limited scalability; cost of antibodies/ligands; may miss EV subpopulations lacking the target marker	[130–132]
Combination approaches (SEC + UF, UC + SEC, SEC + precipitation	Sequential combination of size-exclusion chromatography with ultracentrifugation or polymer-based precipitation	Improved yield and purity compared to SEC alone	More labor-intensive; method standardization still evolving, larger volumes necessary	[54, 121, 123]

EVs extracellular vesicles, UC ultracentrifugation, PEG polyethylene glycol, SEC size-exclusion chromatography, UF ultrafiltration

^a^Ultracentrifugation outcomes are highly dependent on experimental parameters, including rotor type, centrifugal force, and run duration. These variables can promote co-isolation of non-EV particles and vesicle aggregation, thereby influencing downstream proteomic analyses, reproducibility, and cross-study comparability

Overall, no single isolation method consistently delivers both high purity and high yield and method selection must balance these trade-offs against available volume and downstream analytical requirements. A broader comparison including SEC, UC, and multiple commercial kits concluded that the Norgen and Invitrogen kits delivered the best overall performance [115, 116]. In many studies summarized in Table 3, researchers have relied on individual isolation techniques rather than combining multiple techniques.

## Hybrid Isolation Strategies: Combining Strengths

To address these limitations, several studies have explored hybrid isolation strategies that combine methods that demonstrated significant advantages in achieving higher purity and yield (Table 3). When applied to CSF, sequential workflows such as polymer-based precipitation followed by SEC or SEC combined with UC or UF can reduce protein contamination while maintaining EV recovery, especially for larger sample volumes (> 5 mL) [54, 121, 122]. However, these multistep procedures are more labor intensive, though further optimization is needed for small-volume clinical samples [123, 124].

## Emerging Technologies for CSF-Derived EV Isolation

In parallel, emerging technologies have been developed to better accommodate the low-volume, low abundance nature of CSF-derived EVs. Acoustic trapping has emerged as a promising label-free technique that uses sound waves to generate pressure fields that capture and concentrate EVs from CSF. This approach holds potential for improving EV yield while minimizing mechanical damage to the vesicles, which is critical for preserving their structural integrity and functionality in subsequent analyses [66]. Another innovative approach is the ExoGAG method, which leverages the affinity of glycosaminoglycans (GAGs), negatively charged extracellular matrix molecules, to selectively capture EVs through interaction with surface proteins or lipids. This method offers an alternative to traditional isolation techniques and may be particularly attractive for CSF, where starting volumes are restricted and high recovery is critical. However, like other approaches, ExoGAG may face challenges related to the optimization of its specificity and efficiency across diverse sample types, and systematic comparisons of ExoGAG with UC, SEC, and polymer/based workflows in CSF are still required to define its performance for biomarker discovery [133].

Several new methods have been developed to isolate EVs through specific targeting mechanisms, commonly referred to as immuno-isolation [54]. While these methods show promise, their widespread application is limited by the lack of universally accepted biomarkers for selectively isolating EVs from CSF. Common EV markers such as CD9, CD63, and CD81 are widely used for general EV detection but provide no information about the cellular origin. This limitation has prompted interest in markers specific to neurons, oligodendrocytes, or microglia, which could enable more accurate assessment of CNS-derived EVs [134]. L1CAM has often been considered a putative marker for neuronal EVs [64]; however, recent studies have demonstrated that L1CAM is not exclusively associated with EVs and its specificity remains controversial [135–138]. More recently, ATP1A3 has been proposed as an alternative candidate for enrichment of neuronal EVs, based on its neuronal enrichment. However, recent proteinase protection assays suggest that epitopes targeted by commonly used ATP1A3 antibodies may be accessible to protease digestion, indicating that these domains are not protected by the EV lipid bilayer. These findings raise questions regarding the membrane topology and EV association of ATP1A3, underscoring the need for further validation of proposed CNS cell type–specific EV markers [139]. In addition, EVs shed from brain microvascular endothelial cells and choroid plexus epithelium are expected to contribute to CSF-EV pools. Barrier associated proteins such as CLDN5 or PECAM1 on EVs could therefore act as indicators of BBB or blood–CSF barrier status, while at the same time complicating attribution of EV signatures solely to neuronal or glial origin. For oligodendrocyte-derived EVs, commonly cited candidates include myelin oligodendrocyte glycoprotein (MOG), 2′,3′-cyclic nucleotide 3′-phosphodiesterase (CNP), ferritin heavy chain (FTH1), lysosomal-associated membrane protein 2 (LAMP2), myelin basic protein (MBP), and proteolipid protein 1 (PLP1) [140–146]. These markers are frequently detected in EV proteomic analyses of oligodendrocytes and are used to confirm oligodendrocyte origin; however, their specificity varies, and some (notably FTH1) are also present in EVs from other cell types (microglia) [141]. For microglia-derived EVs, candidate markers include TMEM119, CD11b (ITGAM), FTH1, lymphocyte cytosolic protein 1 (LCP1), KCTD12, P2RY12, and TREM2 [49, 64, 139, 144, 147–151]. TMEM119, P2RY12, and TREM2 are considered relatively specific to microglia, whereas CD11b and LCP1 are more broadly expressed in immune cells and can be incorporated into EVs under inflammatory conditions. For astrocyte-derived EVs, markers currently under investigation include integrin alpha-6 (ITGA6) and low-density lipoprotein receptor-related protein 1 (LRP1), which are enriched in astrocyte EV preparations but require further validation to confirm their selectivity [143]. In addition, amyloid precursor–like protein 1 (APLP1) has been proposed as a general CNS-enriched EV marker due to its expression across neural cells and association with brain-derived EVs. While APLP1 can facilitate enrichment of EVs from CNS tissue, it is not restricted to a single cell type and is therefore more accurately considered a CNS-enriched rather than a cell-specific marker [152]. Together, these markers illustrate a spectrum of CNS association: L1CAM and APLP1 are best regarded as CNS-enriched but not strictly neuron-specific, whereas ATP1A3 shows stronger neuronal enrichment but still lacks definitive evidence for exclusive EV association. As a result, none of these candidates currently fulfils the criteria for a robust, neuron-specific EV marker, underscoring the need for further comparative validation across cohorts and platforms. The issue of this biomarker specificity remains a major obstacle and is further discussed later in this section, particularly in the context of EV validation.

## Yield vs Purity: Balancing the Trade-Off in Low-Volume CSF Samples

Based on current literature, either the polymer-based precipitation approach or a hybrid approach appears to yield the most effective results for EV isolation, depending on the available CSF volume. The recommended workflow begins with low-speed centrifugation to effectively remove larger debris, followed by polymer-based precipitation using a commercial kit (e.g., Invitrogen or miRCURY, specific for CSF). This step ensures efficient aggregation of EVs, offering a balance of yield and purity. A subsequent SEC step, using columns with a pore size between 30 and 35 nm, further refines the isolation, by enhancing purity, while maintaining recovery. This integrated method leverages the strengths of each technique while mitigating their individual limitations. As a result, the isolated EVs are better suited for downstream applications, such as proteomic or transcriptomic biomarker studies, where both high purity and sufficient yield are critical. These benefits have mainly been demonstrated in studies using larger CSF volumes, highlighting the need to optimize protocols for smaller clinical samples. A significant limitation of this approach is that it is time consuming, requiring multiple steps and careful optimization of each step in the protocol. Despite this drawback, the benefits of improved purity and yield make it worthwhile, particularly for early biomarker research. Among precipitation-based approaches, ExoGAG offers a newer option that may further improve EV recovery from low volume CSF, although systematic head-to-head comparisons with established kits are still emerging.

## EV Characterization and Validation Standards

Another challenge lies in the characterization and validation of isolated EVs. To ensure that EVs are pure and suitable for the downstream clinical analyses of disease-specific biomarkers (such as those detailed in Table 1), appropriate validation methods must be applied. In this context, EV characterization serves as a prerequisite step that enables reliable measurement of disease-specific biomarkers such as NfL, GFAP, or amyloid-beta/τ in CSF-derived EVs. For example, Western blotting or flow cytometry is first used to confirm the presence of canonical EV markers (e.g., CD9, CD63) in CSF EV preparations, after which targeted immunoassays or LC–MS/MS are applied to quantify the disease-related cargo listed in Table 1 within these validated vesicle fractions [4, 34, 134, 153]. Without proper validation, contaminants or impurities may affect the integrity of the results. This is particularly important given the heterogeneity of CSF-derived EVs, which include a mixture of subtypes. The CSF contains a mix of EV subtypes, such as exosomes, microvesicles, and apoptotic bodies, each with distinct sizes, densities, and molecular compositions [154]. Co-isolation of non-EV components, including lipoproteins and cellular debris, further complicates validation and can adversely affect downstream applications like proteomics or transcriptomics. Proper validation techniques are therefore essential to confirm the purity of the EVs and ensure that they are free from these contaminants. Validation methods are commonly divided into two approaches: (I) assessment of size and morphology and (II) detection of general EV-specific markers [63, 66, 123, 124].

Transmission electron microscopy (TEM) and nanoparticle tracking analysis (NTA) are most frequently used to evaluate EV morphology and size distribution. Several studies have combined TEM and NTA for comprehensive EV characterization. For example, Lopez-Perez et al. (2021) visualized spherical EVs of 30 and 120 nm by TEM, while NTA confirmed a particle size of around 100 nm and identified particle heterogeneity [115]. EV markers, such as CD9 and CD81, were also detected, further confirming vesicle identity. Other study solely used NTA to assess EVs, revealing a homogeneous particle population with an average size of 100 nm. Cryo-TEM was also employed, showing EVs with a spherical shape and smooth surface, consistent with NTA findings.

For marker validation, Western blotting is often used to detect specific EV markers, such as CD9, CD63, and CD81 [5, 155]. One study also included neuron-specific enolase (NSE) and human serum albumin (HSA) as negative controls. However, this study did not include size or morphological validation methods [116, 126], which limits interpretability. A combined validation strategy that integrates size, morphology, and marker expression is therefore essential. However, a lack of standardization in validation methods is evident in the literature. Moreover, recent ISEV guidelines emphasize that distinguishing exosomes from other EV subtypes remains technically unfeasible, adding further complexity to EV validation.

This highlights another critical gap in the field, namely, the lack of understanding of how CSF-derived EVs reflect disease-specific processes. Current studies often focus on bulk EV cargo without distinguishing subpopulations. Emerging single-EV technologies, such as flow cytometry or nanoparticle analysis, should be prioritized to characterize EV heterogeneity and link specific subtypes to clinical phenotypes. Combinatorial marker panels that include neuronal, glial, and endothelial or choroid plexus markers, applied at the single-EV level, will be essential to disentangle parenchymal from barrier-derived vesicle populations and to link specific EV signatures to BBB or blood–CSF barrier dysfunction. Furthermore, identifying EVs derived from specific brain cell types such as neurons, oligodendrocyte, or microglia using validated markers will enhance biological and clinical relevance. Ultimately, successful clinical translation of CSF-derived EVs will require addressing multiple hurdles, including limited reproducibility and lack of standardized isolation and characterization techniques. These inconsistencies hinder the establishment of universal clinical benchmarks for EV-based biomarkers. Furthermore, while advancements in EV isolation are promising, workflows must be optimized for scalability and integration into routine clinical diagnostics. This article does not aim to systematically catalog all published work on CSF-derived EVs but to emphasize conceptual and translational challenges that we consider most critical for advancing the field. Bridging these gaps will necessitate the development of standard protocols and cross-disciplinary collaborations to ensure that EV-based diagnostics can meet clinical needs, aligning with the objectives outlined by the ISEV CSF Task Force [34]. However, standardized isolation is only the first step. Translation of these molecular readouts into a functional understanding of brain drug exposure requires integration of EV data into a quantitative modeling framework.

## From EV Isolation to Application in Physiologically Based Pharmacokinetic Models

CSF-derived EVs offer a noninvasive approach for characterizing the kinetic processes across the BBB and can therefore inform parameters in physiologically based pharmacokinetic (PBPK) models. Isolation of EVs allows quantification of transporter and enzyme mRNA/proteins that are otherwise difficult to access in vivo or in patient populations in which access to tissue samples may be limited or not feasible. Such quantification can provide critical input for PBPK models in these patient populations, enabling the translation of in vitro or ex vivo findings to in vivo [156–161].

The number of EVs released from a given tissue is regulated and can fluctuate due to physiological state, disease, or genetic background. As a result, EV release may not linearly reflect tissue protein abundance. Therefore, it is critical to account for the extent of EV shedding into plasma (or CSF) to avoid bias introduced by differences in EV release. So far, this type of correction has been applied primarily for liver-derived EVs in plasma in different patient populations [159, 160]. Shedding factor is defined as a correction factor that adjusts EV-associated protein levels using tissue-specific markers. It aims to normalize EV-associated protein levels for between-patient variabilities in the rate of shedding, hence enabling more reliable extrapolation to tissue expression. Current evidence based on liver plasma EVs indicates variability in EV shedding factors across different populations [159, 160]. Importantly, these liver EV studies demonstrate that shedding factor–corrected EV gene levels can align with tissue protein abundance, providing a proof of concept for applying this framework to other tissues, including the brain. EV abundance data corrected by shedding factors for a number of hepatic transporters and enzymes have provided individualized characterization of drug elimination pathways; these have been integrated into PBPK models for patients with renal impairment [161] or cardiovascular diseases [162]. Plasma EV-informed expression data for ADME-relevant hepatic enzymes and transporters have also been used to capture population heterogeneity and support dose individualization [159, 161]. However, to date, no studies have applied CSF-derived EVs to inform PBPK modeling of brain exposure or to investigate interindividual variability in the expression of PK and pharmacodynamic (PD) proteins in the brain under disease conditions. Liver plasma EV studies provide empirical evidence that shedding factors can align EV protein levels with tissue protein abundance. However, applying this approach to CSF-derived brain EVs remains untested and should therefore be regarded as a conceptual extension of this framework.

EV-derived data can therefore provide complementary information to tissue-derived abundance measurements, which provide a direct quantitative measure of transport expression within the BBB [163]. Together, EV- and tissue-based expression data may establish a coherent framework linking circulating biomarkers to tissue expression, addressing interpatient variability and enhancing mechanistic modeling of brain exposure, based on pathophysiology and biology as a complement to any imaging or EEG (electroencephalogram) information [164].

Proteomics-based quantification methods such as QconCATs-based LC/MS/MS are key tools for generating the quantitative protein data in different tissues and patient populations to inform PBPK models [163, 165–167]. QconCATs use synthetic proteins containing unique peptides as internal standards, enabling absolute quantification of transporters and drug-metabolizing enzymes in BBB and other tissues. The same targeted panels can, in principle, be applied to EV-derived proteomes to correlate circulating and tissue expression profiles. In this context, QconCAT-informed absolute abundance data for ADME-relevant proteins in tissues provide the required reference against which EV-associated levels are scaled (e.g., via shedding factors), thereby quantitatively linking liquid biopsy readouts to mechanistic PBPK models in defined patient populations [168]. Although current applications focus primarily on pharmacokinetics, integrating pharmacodynamic markers into EV-based analyses could further strengthen the link between drug exposure, efficacy, and molecular responses. Crucially, the same biological mechanisms that allow us to “read” the brain’s state through EVs can be reverse-engineered to “write” therapeutic instructions, transitioning the role of these vesicles from diagnostic sensors to targeted delivery vehicles.

## EVs as Vehicles for CNS Drug Delivery

EVs are increasingly recognized as promising biological alternatives to synthetic drug delivery systems. Their endogenous biological origin, inherent natural ability to cross the BBB, and capacity to carry various biomolecules make them particularly attractive for targeted drug delivery in the brain [169]. From a therapeutic perspective, EV-based interventions fall into two main categories: (I) leveraging their intrinsic biological functions to modulate disease process and (II) engineering them as delivery vehicles for therapeutic agents.

### Intrinsic Biological Activity of EVs

The intrinsic biological functions of EVs, includes intercellular signaling and immunomodulation, to influence tissue repair or pathological processes [170]. MSC-derived EVs licensed with IFN-γ and TGF-β1 actively reprogram macrophages and expand regulatory T cells, revealing that EVs can coordinate complex immune responses through combined modulation of cytokine release and cell phenotype [171]. Similarly, hiPSC-NSC-EVs attenuate amyloid beta-42 oligomer–induced neuronal degeneration by concurrently reducing oxidative stress, restoring mitochondrial and autophagy function and limiting tau phosphorylation, demonstrating that EVs can simultaneously engage multiple, interdependent cellular pathways [172]. Collectively, these findings show that EVs mediate therapeutic effects through their intrinsic biological functions, modulating multiple interconnected cellular pathways rather than acting through a single mechanism, reflecting their inherent mechanistic complexity. This multitarget therapeutic capacity is particularly relevant for neurodegenerative diseases where pathology involves interconnected molecular cascades such as amyloid-β accumulation, tau pathology, neuroinflammation, and mitochondrial dysfunction suggesting that EV-based therapies may address disease complexity more effectively than single-target approaches.

### Engineered EVs for Targeted Therapeutics

The second strategy utilizes EVs as drug delivery vehicles, in which they are engineered or loaded with therapeutic agents, such as nucleic acids, small molecules, or chemotherapeutics, for targeted delivery to specific cells or tissues, a process that introduces additional technical challenges compared to the first approach [173]. For this approach, EVs can be loaded with their cargo using either preisolation (preloading) or post-isolation (post-loading). During biogenesis, preloading involves modifying donor cells to package the desired cargo naturally into secreted EVs. In contrast, post-loading involves incorporating the cargo into EVs after isolation using physical or chemical methods like electroporation, sonication, or incubation. Currently, there is no agreement on which approach is superior, as each has distinct benefits and limitations. Preloading, however, tends to have minimal effects on EV structure and composition, thereby preserving their natural properties, including targeting ability and biodistribution. As an example of preloading, photoporation has been used to deliver compounds into the cytosol of human embryonic kidney 293 T cells expressing enhanced green fluorescent protein (HEK293T-EGFP). This process generated EVs with up to 53% of the population actively loaded when using the EV-targeting molecule NBAF (Alexa Fluor 647), compared with 12% with passive AF10 molecules. Importantly, EVs maintained their size, ultrastructure, and marker expression, demonstrating that cytosolic photoporation can efficiently generate functional EVs with defined payloads [174]. In contrast, the post-loading approach requires prior isolation of EVs and careful characterization to determine their subtypes and biogenesis (e.g., exosomes vs. microvesicles), which remains a major challenge in EV research, as discussed in the next section. For example, CD81-positive extracellular vesicles were first enriched using antibody-coated magnetic beads and subsequently loaded with microRNA via electroporation. This strategy enabled rapid, subpopulation-specific cargo incorporation while preserving EV membrane integrity and surface proteins and reduced purification time compared to conventional ultracentrifugation. Post-loading allows precise control over cargo introduction without altering parent cells and facilitates optimization for different molecules, but it can be limited by variable loading efficiency, potential membrane disruption, and the need for careful removal of unincorporated cargo [175].

Together, these concrete examples demonstrate that both pre- and post-loading strategies can generate EVs that retain their intended biological activity, including proper targeting, cargo delivery, and modulation of recipient cell pathways. However, developing reliable therapeutic applications will require a thorough understanding of how cargo loading affects EV formation, membrane properties, and uptake by target cells, as well as standardized methods to ensure reproducible EV composition, such as size, surface protein profile, cargo content, and consistent functional performance in terms of delivery efficiency and biological effect across production batches. Nevertheless, the clinical translation of these therapeutic platforms remains essential upon a definitive correlation analysis between CSF-EV cargo and the parent brain tissue. Determining the confidence with which EV signatures mirror the brain proteome is a fundamental step for ensuring both diagnostic accuracy and therapeutic safety.

## Required Additional Work to Make Better Use of EVs

**Fig. 3:**
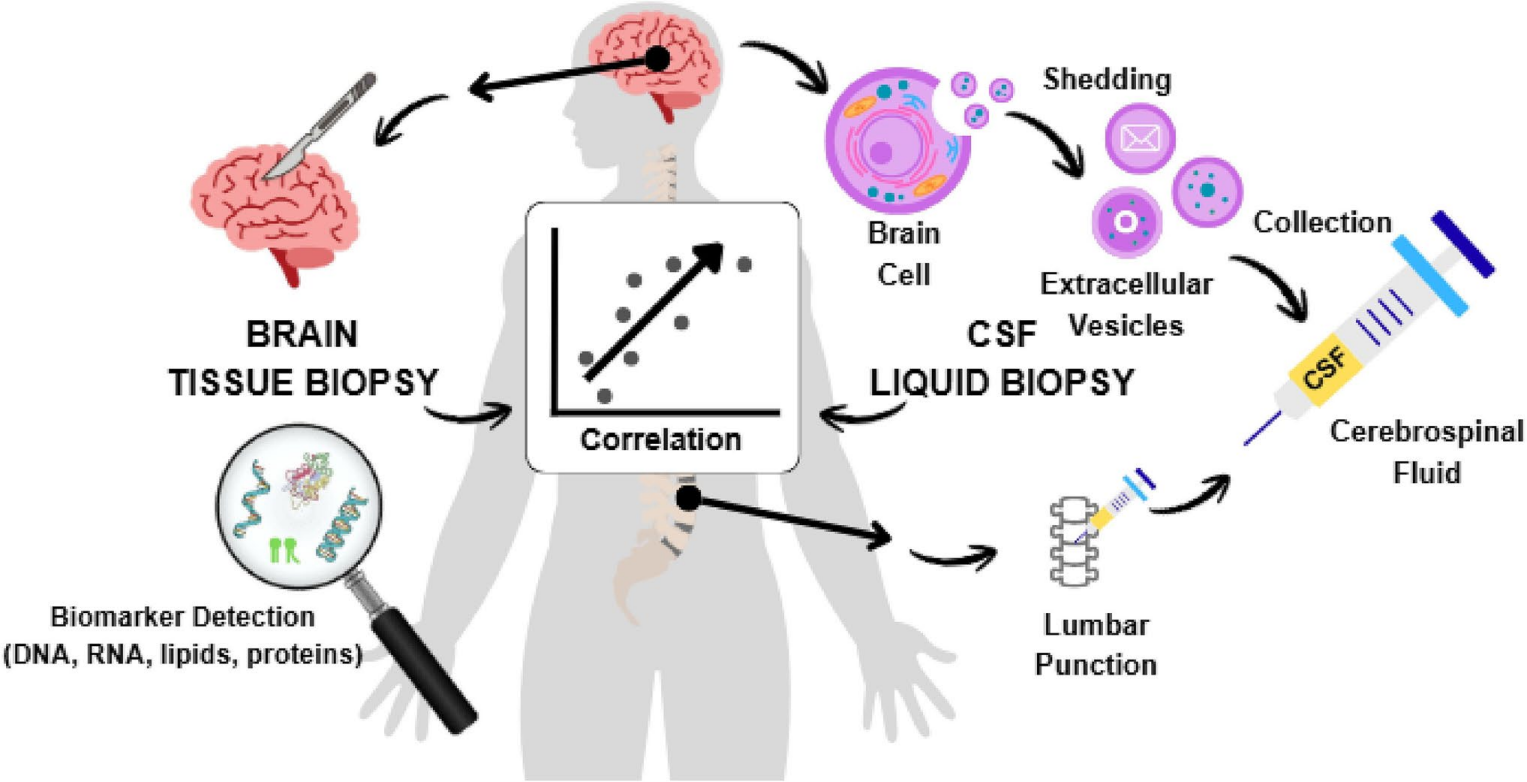
Conceptual framework for correlating brain tissue biopsies with cerebrospinal fluid liquid biopsy in future studies (CSF = cerebrospinal fluid)

The use of CSF-derived EVs as biomarkers for CNS diseases requires an established correlation between EV-associated molecular signals and corresponding changes in brain tissue (Fig. 3). Without this relationship, it remains difficult to determine how EV-derived measurements relate to the magnitude and direction of molecular alterations occurring within the brain. Implementing experimental designs based on matched samples, in which brain tissue and CSF from the same individuals are analyzed in parallel, is therefore essential. Recent studies have demonstrated that CSF-derived EV cargo can reflect and even propagate brain pathology, highlighting the relevance of such correlations [49, 66, 114, 176]. Establishing correlations between these matching datasets represents a foundational step for meaningful interpretation of EV-derived signals (DNA, RNA, protein, lipids), including the establishment of a shedding factor as discussed in the section of PBPK models. Once such relationships are defined, they will enable quantitative inference, allowing measurements obtained from CSF-derived EVs to be translated into estimates of molecular changes within the brain itself. This step is critical for advancing EV-based liquid biopsies from qualitative indicators toward quantitative biomarkers of CNS pathology as multiple biological factors. This transition is fundamentally tied to our ability to quantify confounding factors such as BBB dysfunction.

## BBB Disruption and EV Signatures

Disease-specific BBB alterations dictate the diagnostic utility of EVs: In the context of glioblastoma, EVs facilitate active barrier disruption, whereas in AD and MS, they provide a noninvasive readout of underlying endothelial and inflammatory pathology. Given their ability to mirror BBB alterations, EVs emerge as promising biomarkers for monitoring vascular integrity in CNS diseases. Integrating CSF-derived EV analyses with matched brain tissue provides mechanistic context for the analysis of BBB disease-related changes and dysfunction. If performed, such studies could enable direct correlation of molecular changes observed in CSF-derived EVs with those occurring in brain tissue, thereby improving our understanding of how BBB dysfunction contributes to CNS pathology. Microvessels express a range of clinically and therapeutically relevant transporter proteins [177, 178]. Linking these microvessel measurements to CSF-EV molecular profiles can connect EV cargo with BBB alterations and EV shedding into the CSF, enabling the identification of disease-specific EV signatures. This approach may help distinguish subtle BBB disruptions, capture disease heterogeneity, and provide a more precise biomarker for monitoring disease progression.

## Future Perspectives

While most studies have primarily focused on nucleic acids and proteins, there is growing interest in expanding the focus to lipids. Many CNS diseases are associated with changes in lipid content, making lipidomic analysis an important avenue for further investigation. However, only a limited number of studies have explored lipidomics in extracellular vesicles, highlighting an emerging opportunity for future research to deepen our understanding of lipid-related changes in neurological disorders [179–184].

Beyond conventional omics approaches, integrating spatial omics such as spatial proteomics and lipidomics represents an important frontier in EV research. These technologies can spatially resolve the distribution of molecules such as lipids, proteins, and nucleic acids within and even potentially around EVs, revealing their localization and potential functional roles. Specifically, it would be valuable to determine whether these molecules are part of the internal cargo of the EVs or if some are localized on the EV membrane. However, as EVs are nanoscale particles suspended in solution, current spatial proteomics and lipidomics methods often lack the resolution to clearly differentiate membrane proteins from luminal ones. Techniques like secondary ion mass spectrometry (SIMS) and other emerging innovations, which achieve nanoscale resolution on the order of ~ 200 nm, offer promising avenues to overcome these limitations [185, 186]. Although EVs are typically around 30 nm, smaller than the current resolution of these methods, ongoing advances may soon enable more precise spatial characterization at this scale. Ultimately, these technologies could allow detailed molecular mapping of EVs, providing novel insights into their functions and roles in CNS processes and disorders.

As an alternative to spatial omics, selective surface labeling strategies such as membrane-impermeant biotinylation followed by mass spectrometry have shown promise in characterizing the EV surface proteome (surfaceome). This method has been applied to large EVs (mean diameter of 310 nm), enabling the identification of more than 900 surface-accessible proteins, including receptors and cytoskeleton-associated components, while preserving vesicle integrity [187]. This understanding would also enhance the ability to unravel the correlation between EVs and the BBB. For example, molecules attached to the EV membrane could play a direct role in modulating the integrity or permeability of the BBB, potentially influencing its stable function or contributing to pathological changes. By identifying such membrane-associated interactions, researchers could better elucidate the mechanisms by which EVs mediate signaling between the central nervous system and the periphery.

These insights would aid the development of pharmacological models by identifying specific EV-associated molecules or interactions that directly influence BBB permeability and stability. For instance, incorporating quantitative data on how EV cargo modulates tight junction proteins, transporter activity, or signaling pathways in endothelial cells enables more precise modeling of BBB function. This allows researchers to simulate how changes in EV composition or abundance may alter drug penetration into the CNS.

From a pharmacodynamic perspective, EV-associated molecules could serve as functional biomarkers of drug response, reflecting cellular events at the BBB or within the brain environment. Integrating EV-derived data into PD models provides a promising avenue to link drug concentrations to therapeutic outcomes more accurately, enhancing predictions of efficacy and toxicity by detecting, for example, early molecular signatures of neuroinflammation or cellular stress responses. Importantly, accessing this information from human biofluids such as CSF or blood could reduce dependence on animal models in early PK/PD studies. If established, this approach may improve translational accuracy and contribute to ethical research practices by supporting the 3Rs principle, refining, reducing, and ultimately replacing animal use where feasible.

Moving forward, standardization and reproducibility remain critical challenges. Future work should focus on harmonized EV isolation and characterization protocols for CSF to ensure high purity, consistency, and scalability. Comparative studies across isolation techniques are needed to identify optimal methods for specific downstream applications. Additionally, as CSF contains various EV subtypes, including exosomes, microvesicles, and apoptotic bodies, future studies should investigate how these subtypes differ in their molecular cargo and roles in neurological processes. Identifying reliable specific markers or surface proteins to distinguish these subtypes could provide deeper insights into their functions and therapeutic potential.

Overall, future research should focus on the roles of EVs in CNS diseases by examining how their molecular cargo changes in response to these conditions, potentially identifying biomarkers for diagnosis, monitoring, but also mechanistic understanding. Additionally, EVs offer promising potential as drug delivery vehicles across the BBB, with efforts directed at engineering EVs to carry therapeutic molecules and enhancing their uptake by target cells.

## Conclusion

EVs hold exceptional promise for biomarker discovery and therapeutic innovation in CNS research. CSF-derived EVs offer the possibility of a direct readout of central nervous system processes. Their molecular cargo comprising proteins, nucleic acids, and lipids presents an opportunity to gain valuable insights into both healthy and diseased brains, without the need for invasive tissue biopsies. Beyond their value as biomarkers, EVs offer a unique opportunity to address the challenge of limited direct brain access for CNS-targeted therapies. CSF-derived brain EVs may provide quantifiable protein level data that could be integrated into pharmacokinetic and pharmacodynamic models. Ultimately, liquid biopsy–informed PBPK/PD models, particularly those incorporating CSF-derived EV data, are envisaged to support predictions of effective CNS drug concentrations, guide dosing regimens, and improve understanding of target engagement in CNS drug development. Integrating these multidisciplinary efforts will transform CSF-derived EVs from promising biomarkers into powerful tools for diagnosis, mechanistic insight, and therapeutic innovation in CNS disorders. Overall, our perspective highlights CSF-derived EVs as a central, noninvasive CNS-specific liquid biopsy platform, with the potential to complement imaging and tissue data to advance biomarker discovery, BBB characterization, and PBPK-informed precision dosing in neurological diseases.

## Data Availability

No datasets were generated or analyzed during the current study.

## Declarations

### Additional Information

Clinical trial number: not applicable.
